# Isocitrate dehydrogenase 2 mutation is a frequent event in osteosarcoma detected by a multi-specific monoclonal antibody MsMab-1

**DOI:** 10.1002/cam4.149

**Published:** 2013-10-17

**Authors:** Xing Liu, Yukinari Kato, Mika Kato Kaneko, Masato Sugawara, Satoshi Ogasawara, Yuta Tsujimoto, Yasushi Naganuma, Mitsunori Yamakawa, Takashi Tsuchiya, Michiaki Takagi

**Affiliations:** 1 Department of Regional Innovation, Tohoku University Graduate School of Medicine2-1 Seiryo-machi, Aoba-ku, Sendai, Miyagi, 980-8575, Japan; 2 Department of Orthopaedic Surgery, Yamagata University School of Medicine2-2-2 Iida-nishi, Yamagata, 990-9585, Japan; 3 Department of Diagnostic Pathology, Yamagata University School of Medicine2-2-2 Iida-nishi, Yamagata, 990-9585, Japan

**Keywords:** Isocitrate dehydrogenase 1, isocitrate dehydrogenase 2, monoclonal antibody, mutations, osteosarcoma

## Abstract

Somatic mutations of isocitrate dehydrogenase (IDH) 1 and IDH2 occur in gliomas, acute myeloid leukemia, and cartilaginous tumors. Somatic mosaic IDH1/2 mutations are also reported in Ollier disease and Maffucci syndrome, which are characterized by multiple central cartilaginous tumors. Although IDH1/2 mutation analysis against osteosarcoma has been performed in several studies, no IDH1/2 mutation has been reported. Herein, we newly report the IDH2-R172S mutation in three of 12 (25%) osteosarcoma patients, which was detected by direct DNA sequencing. No monoclonal antibody (mAb) has been reported against IDH2-R172S mutation. However, we demonstrate that the IDH2-R172S peptide was recognized by our established multi-specific anti-mutated IDH1/2 mAb, MsMab-1, in enzyme-linked immunosorbent assay. Western blot analysis revealed that MsMab-1 reacts with PA tag combined recombinant proteins of IDH2-R172S. Furthermore, MsMab-1 stained IDH2-R172S-expressing osteosarcoma tissues in immunohistochemistry. The MsMab-1 stained nine of 32 (28.1%) osteosarcomas in a tissue microarray. This report is the first describing IDH2 mutations in osteosarcoma, which can be detected by MsMab-1 mAb. Taken together, these results show that MsMab-1 can be anticipated for use in immunohistochemical determination of IDH1/2 mutation-bearing osteosarcoma.

## Introduction

Although osteosarcoma is the most common primary malignant bone tumor in children and young adults, the advance of aggressive systemic chemotherapy has improved the survival rate for osteosarcomas. Nevertheless, the survival rate of osteosarcoma patients with primary lung metastases remains poor compared to that of patients with localized disease [1]. Moreover, multidrug combination chemotherapy for osteosarcoma entails ototoxicity, cardiac toxicity, and secondary malignancies [2]. To resolve these problems, it is expected to be necessary to develop molecular targeted agents with high tumor specificity.

Somatic mutations of isocitrate dehydrogenase (IDH) 1 and IDH2 were first found in gliomas [3] to convert α-ketoglutarate to oncometabolite R(-)-2-hydroxyglutarate (2-HG), although IDH1 and IDH2, respectively, catalyze the oxidative carboxylation of isocitrate to α-ketoglutarate in cytosol and mitochondria [4]. Results of 2-hydroxyglutarate dehydrogenase deficiency show that 2-HG accumulates in association with the inherited metabolic disorder 2-hydroxyglutaric aciduria because 2-hydroxyglutarate dehydrogenase changes 2-HG to α-ketoglutarate [5]. Patients with 2-hydroxyglutarate dehydrogenase deficiencies are known to face increased risk of brain tumors because they accumulate 2-HG in the brain and develop leukoencephalopathy. Furthermore, elevated 2-HG levels in the brain contribute to increased risk of cancer by increasing reactive oxygen species (ROS) concentrations [6]. IDH1/2 mutations were also reported in acute myeloid leukemias (AML) [7] and cartilaginous tumors [8–10]. Kerr et al. [11] also reported that IDH1/2 mutations were detected in chondrosarcoma, but not in chondroblastic osteosarcomas, suggesting that mutation analysis of IDH1/2 has yielded a promising biomarker for distinguishing chondrosarcoma from chondroblastic osteosarcoma.

The IDH1 mutations are remarkably specific to a single codon in the conserved and functionally important arginine 132 residue (R132). In contrast, the IDH2 mutations are specific to a single codon in arginine 172 residue (R172). IDH2 mutations of AML were discovered subsequently in arginine 140 residue (R140), which is found more frequently than R172 [12]. Most changes by far are heterozygous. In gliomas, IDH1 mutations were reported as IDH1-R132H (664/716: 92.7%), IDH1-R132C (29/716: 4.2%), IDH1-R132S (11/716: 1.5%), IDH1-R132G (10/716: 1.4%), and IDH1-R132L (2/716: 0.2%) [13]. We established IDH1-R132H-specific monoclonal antibodies (mAbs) HMab-1 [14]/IMab-1 [15], IDH1-R132S-specific mAb SMab-1 [16], and IDH1-R132G-specific mAbs GMab-r1 [17]/GMab-m1 [18], although another group reported only one mAb against IDH1-R132H [19]. In contrast, IDH2 mutations were reported as IDH2-R172K (20/31: 64.5%), IDH2-R172M (6/31: 19.3%), and IDH2-R172W (5/31: 16.2%) in gliomas [13]. To date, we have established IDH2-R172K-specific mAb KMab-1 [20], IDH2-R172M-specific mAb MMab-1 [20], and IDH2-R172W-sepcific WMab-1 [21]. A recent report has described multi-specific anti-mutated IDH1/2 mAbs, MsMab-1 [22] and MsMab-2 [23], which are useful for diagnosis of IDH1/2 mutation-bearing gliomas. This article describes the IDH2-R172S mutation in 3 of 12 (25%) osteosarcoma patients, which was detected by direct DNA sequencing. Furthermore, MsMab-1 stained nine of 32 (28.1%) osteosarcomas in a tissue microarray.

## Material and Methods

### Cell lines and tissues

Chinese hamster ovary (CHO)-K1 and U-2 OS osteosarcoma cell line were obtained from the American Type Culture Collection (ATCC, Manassas, VA) and were cultured at 37°C in a humidified atmosphere of 5% CO_2_ and 95% air in RPMI 1640 and Dulbecco's modified Eagle medium (DMEM), respectively, including 2 mmol/L l-glutamine (Nacalai Tesque Inc., Kyoto, Japan) supplemented with 10% heat-inactivated fetal bovine serum (FBS; Life Technologies Inc., Carlsbad, CA). This study examined 12 osteosarcoma patients who underwent surgery at Yamagata University. The ethical committee of the Yamagata University Faculty of Medicine approved this study. Informed consent for obtaining samples and for subsequent data analyses was obtained from each patient or the patient's guardian. Tissue microarrays of osteosarcomas were purchased from Cybrdi Inc. (Frederick, MD). The pathological diagnosis of all specimens in this study was confirmed by a pathologist (Prof. Mitsunori Yamakawa, Yamagata University School of Medicine).

### Direct DNA sequencing of IDH1 and IDH2 mutations and subcloning

Genomic DNA was extracted from formalin-fixed, paraffin-embedded tissue sections using MightyAmp for FFPE (Takara Bio Inc., Shiga, Japan) according to the manufacturer's instructions. Polymerase chain reaction (PCR) primers for the genomic region corresponding to IDH1 exon 4, which encodes codon R132, and the flanking intronic sequences were the following: human IDH1 sense (5′-CGGTCTTCAGAGAAGCCATT-3′) and human IDH1 antisense (5′-GCAAAATCACATTATTGCCAAC-3′). PCR primers for the genomic region corresponding to IDH2 exon 4, which encodes codon R172, and the flanking intronic sequences were the following: human IDH2 sense (5′-CAAGCTGAAGAAGATGTGGAA-3′) and human IDH2 antisense (5′-CAGAGACAAGAGGATGGCTA-3′). The PCR conditions were 98°C for 2 min (one cycle), followed by 40 cycles of 98°C for 10 sec, 60°C for 15 sec, 68°C for 30 sec, and extension at 68°C for 10 min with MightyAmp DNA polymerase (Takara Bio Inc.). Cycle sequencing was conducted using the sequencing primer for IDH1 (5′-CCATTATCTGCAAAAATATC-3′) and for IDH2 (5′-AGCCCATCATCTGCAAAAAC-3′). Subsequently, the PCR products were subcloned into pCR4-TOPO vectors (Life Technologies Inc.), after which 20 clones were sequenced to confirm the R132/R172 mutations [24].

### Enzyme-linked immunosorbent assay

Enzyme-linked immunosorbent assays (ELISAs) were performed as previously described [22].

### Plasmid preparation

Human IDH1 cDNA (GenBank accession no. AF113917 or BC012846) and human IDH2 cDNA (accession no. NM_002168) encoding a full-length open reading frame (ORF) were obtained using PCR with a human lung cDNA library (Cosmobio Co. Ltd., Tokyo, Japan) and cDNA derived from the U373 glioblastoma cell line as template, respectively. The following primer set was used for IDH1: EcoRI-IDH1-F1, 5′-cacgaattcATGTCCAAAAAAATCAGTGG-3′ and SalI-IDH1-R1, 5′-gtggtcgacTTAAAGTTTGGCCTGAGCTA-3′. The following primer set was used for IDH2: EcoRI-IDH2.F1, 5′-ccgaattcgggATGGCCGGCTACCTGCGGG-3′ and SalI-IDH2wterR1359, 5′-gccgtcgacCTACTGCCTGCCCAGGGCTCT-3′. The amplified cDNA was subcloned into a pcDNA3.1/V5-His-TOPO vector (Life Technologies Inc.). Substitution of the arginine 132 (R132) to appropriate amino acid codons in IDH1 or arginine 172 (R172) to appropriate amino acid codons in IDH2 was done using the QuikChange Lightning site-directed mutagenesis kit (Agilent Technologies Inc., Santa Clara, CA). The full-length IDH2 and each mutated ORF were amplified using the primer set: EcoRI-IDH2.F1 and IDH2woterR1356-XhoI, 5′-taactcgagcgCTGCCTGCCCAGGGCTCTG-3′. These PCR products were digested with EcoRI and XhoI restriction enzymes, and were subcloned into pcDNA3 vector (Life Technologies Inc.) together with the nucleotide sequence (ctcgagTGGCGTTGCCATGCCAGGTGCCGAAGATGATGTGGTGTAAtctaga), which encodes 12 amino acids, GVAMPGAEDDVV (PA tag) [20–22].

### Protein expression using mammalian cells

CHO cells were transfected with appropriate amounts of plasmids, pcDNA3.1/IDH1-WT, pcDNA3.1/IDH1-R132H, pcDNA3.1/IDH1-R132C, pcDNA3.1/IDH1-R132S, pcDNA3.1/IDH1-R132G, pcDNA3.1/IDH1-R132L, pcDNA3/IDH2-WT, pcDNA3/IDH2-R172K, pcDNA3/IDH2-R172M, pcDNA3/IDH2-R172W, pcDNA3/IDH2-R172G, or pcDNA3/IDH2-R172S using Lipofectamine LTX (Life Technologies Inc.) according to the manufacturer's instructions. U-2 OS osteosarcoma cells were transfected with appropriate amounts of plasmids, pcDNA3/IDH2-WT and pcDNA3/IDH2-R172S using ScreenFect A (Wako Pure Chemical Industries Ltd., Osaka, Japan) according to the manufacturer's instructions. The expression level of IDH1/2 was confirmed using Western blot analyses. The morphology of transfected cells was observed using FLoid Cell Imaging Station (Life Technologies Inc.). The growth of transfected cells was analyzed using TC10 automated cell counter (Bio-Rad Laboratories Inc., Philadelphia, PA). The cell migration was detected by scratch assay as described previously [25].

### Western blot analyses

Cultured cell pellets were lysed with RIPA buffer (Thermo Fisher Scientific Inc., Rockford, IL) for 30 min on ice. The lysate supernatants were centrifuged for 15 min at 20,630 × g to remove cellular debris. Cell lysates containing 10 μg of total protein were prepared for Western blot analyses by boiling in sodium dodecyl sulfate (SDS) sample buffer including 2-mercaptoethanol (2-ME; Nacalai Tesque Inc.). They were electrophoresed on 5–20% polyacrylamide gels (Wako Pure Chemical Industries Ltd.). The separated proteins were subsequently transferred to a polyvinylidene difluoride (PVDF) membrane (EMD Millipore Corp., Billerica, MA). After blocking with 4% skim milk in phosphate-buffered saline (PBS) with 0.05% Tween 20 for 15 min, the membrane was incubated with 1 μg/mL of MsMab-1 [[22]**]**, MsMab-2 [[23]**]**, anti-PA tag (NZ-1) [[20–22]**]**, anti-V5 tag (MBL Co. Ltd., Nagoya, Japan), anti-IDH2 (5F11; Sigma-Aldrich, St. Louis, MO), and anti-IDH1 (RcMab-1) [[22]**]** for 30 min. Then the membrane was incubated with peroxidase-conjugated secondary antibodies (1:1000 diluted; Dako, Glostrup, Denmark) for 15 min, and developed with ImmunoStar LD Chemiluminescence Reagent (Wako Pure Chemical Industries Ltd.) using a Sayaca-Imager (DRC Co. Ltd., Tokyo, Japan).

### Immunohistochemical analyses

Mutated IDH2-R172S protein expression was determined immunohistochemically in paraffin-embedded tumor specimens. No osteosarcoma specimen was decalcified in the osteosarcoma patients of Yamagata University. Briefly, 4-μm-thick histologic sections were deparaffinized in xylene and rehydrated. Then, they were autoclaved in citrate buffer (pH 6.0; Dako) for 20 min. Sections were incubated with 5 μg/mL of MsMab-1 overnight at 4°C, followed by treatment with an LSAB kit (Dako). Color was developed using 3,3-diaminobenzidine tetrahydrochloride (DAB; Dako) for 10 min, and counterstained with hematoxylin. Serial sections were used for hematoxylin-eosin staining. MsMab-1 staining was assessed semi-quantitatively from the percentage of tumor cells with cytoplasmic staining: 0, no staining; +, <10%; ++, 10–50%; and +++, >50%, and staining intensity: −, no staining; +, weak; ++, medium; +++, strong.

## Results

### Mutational analysis of IDH1/2 in osteosarcoma

**Figure 1:**
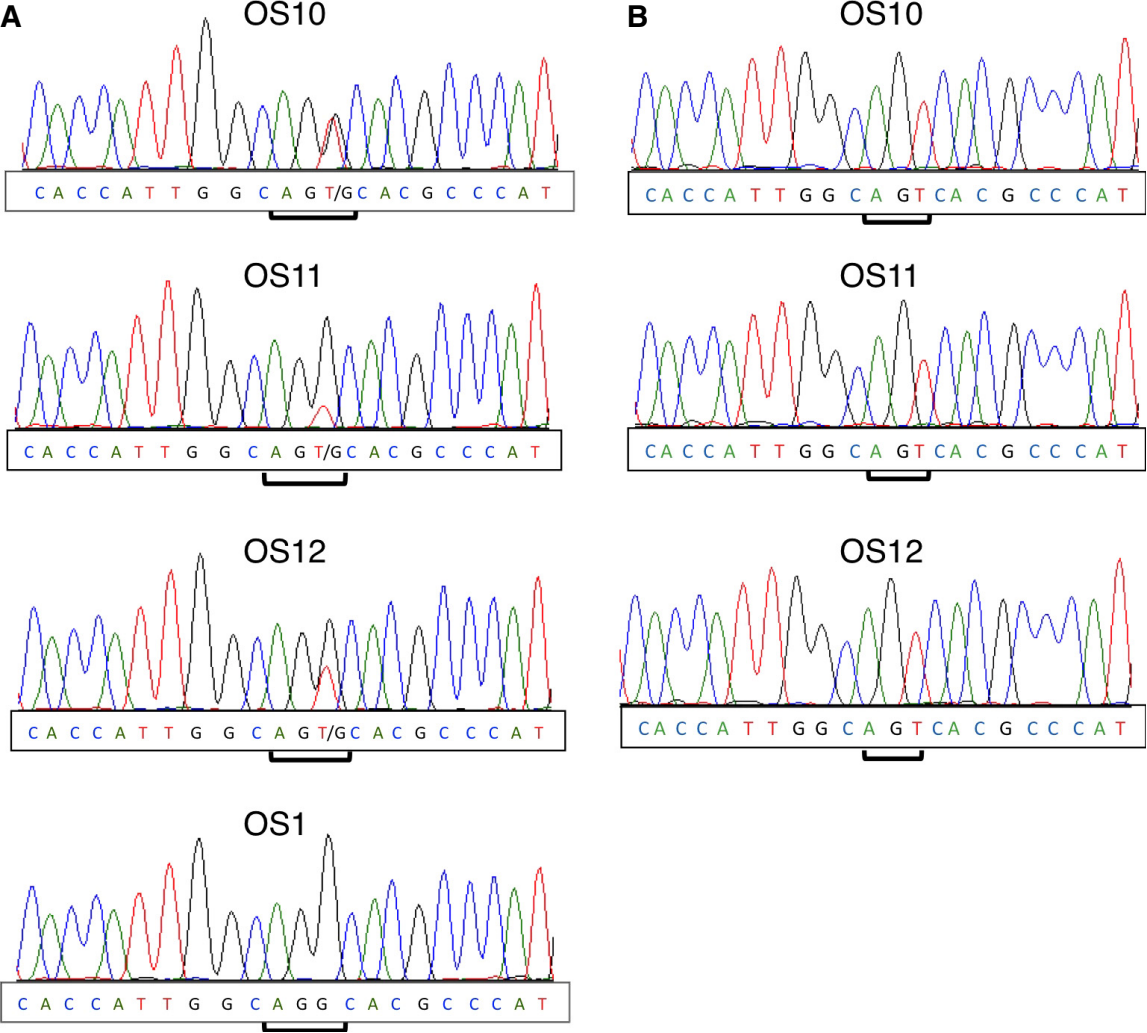
Mutational analysis of IDH1/2 in osteosarcoma. (A) DNA direct sequencing was performed against OS10, OS11, OS12, and OS1. (B) PCR products of OS10, OS11, and OS12 were subcloned into pCR4-TOPO vectors. Each clone was sequenced to confirm the IDH2-R172 mutation. IDH, isocitrate dehydrogenase; PCR, polymerase chain reaction.

IDH1/2 mutation analysis against osteosarcoma has been performed in several studies [8, 11], but no IDH1/2 mutation has been reported yet. For this study, we analyzed IDH1 and IDH2 mutations using 12 osteosarcoma specimens with direct DNA sequencing (Table 1). All samples were primary tumors obtained by biopsy before chemotherapy or surgery. Of the 12 patients, 2 showed lung metastasis before chemotherapy. In accordance with previous reports, no IDH1 mutation was observed in 12 samples. In contrast, three of 12 (25%) osteosarcoma samples, two of which were osteoblastic osteosarcoma (OB) and one of which was high-grade surface osteosarcoma (HGS), possessed IDH2 mutations. Two OB patients with IDH2 mutation showed grade 3 histological necrosis after preoperative chemotherapy. Continuous disease-free survival was assessed for all three patients with IDH2 mutations (75–128 months). It is noteworthy that all three IDH2 mutations were of IDH2-R172S (AGG>AGT; Fig. 1A), which is also frequently observed in chondrosarcomas [8]. To confirm this result, we performed subcloning of these three PCR products. As presented in Figure 1B, all three cases included the IDH2-R172S mutant sequence (OS10, 14/16 (87.5%); OS11, 2/17 (11.8%); and OS12, 6/17 (35.3%)), demonstrating that all three osteosarcoma samples include IDH2-R172S mutations.

**Table 1:** Clinicopathological characteristics of osteosarcoma samples

Alias	Age	Gender	Site	Sample type	Sample class	Diagnosis	Grade^1^	Metastasis^2^	Status	IDH1	IDH2	MsMab-1	

												Percentage^3^	Intensity^4^
OS1	22	F	Tibia	Biposy	Primary	OB	Grade 0	−	CDF	Wild type	Wild type	−	−
OS2	16	M	Humerus	Biposy	Primary	OB	Grade 0	+	DOD	Wild type	Wild type	−	−
OS3	12	M	Tibia	Biposy	Primary	OB	Grade 3	−	CDF	Wild type	Wild type	−	−
OS4	56	F	Femur	Biposy	Primary	OB	Grade 1	−	CDF	Wild type	Wild type	−	−
OS5	80	M	Mandible	Biposy	Primary	OB	Grade 0	−	DOD	Wild type	Wild type	−	−
OS6	16	M	Humerus	Biposy	Primary	CB	Grade 0	−	CDF	Wild type	Wild type	−	−
OS7	14	F	Humerus	Biposy	Primary	OB	Grade 2	+	NED	Wild type	Wild type	−	−
OS8	10	M	Tibia	Biposy	Primary	CB	Grade 1	−	CDF	Wild type	Wild type	−	−
OS9	71	M	Mandible	Biposy	Primary	OB	No chemotherapy	−	CDF	Wild type	Wild type	−	−
OS10	6	M	Femur	Biposy	Primary	OB	Grade 3	−	CDF	Wild type	R172S	++	++
OS11	10	M	Tibia	Biposy	Primary	OB	Grade 3	−	CDF	Wild type	R172S	+	++
OS12	29	M	Femur	Biposy	Primary	HGS	Grade 0	−	CDF	Wild type	R172S	−	−

OB, osteoblastic osteosarcoma; CB, chondroblastic osteosarcoma; HGS, high-grade surface osteosarcoma; CDF, continuous disease free; DOD, dead of disease; NED, no evidence of disease.

1 Histological necrosis after preoperative chemotherapy; Grade 0, 0–50%; Grade 1, 51–90%; Grade 2, 91–99%; Grade 3, 100%.

2 Lung metastasis existed before chemotherapy.

3 −, no staining; +, <10%; ++, 10–50%; and +++, >50%.

4 −, no staining; +, weak; ++, medium; +++, strong.

### Specificity of MsMab-1 against IDH1/2 mutants in ELISA and Western blot analyses

We recently established a multi-specific anti-mutated IDH1/2 mAb, MsMab-1 [22]. Although MsMab-1 reacts with several IDH1 mutations such as IDH1-R132H, IDH1-R132S, and IDH1-R132G, the reaction of MsMab-1 against other IDH1 mutants or IDH2 mutants has not been clarified. For this aim, we investigated the reactivity of MsMab-1 mAb against 20 peptides of IDH1 mutation and 20 peptides of IDH2 mutation (Table 2). As shown in Tables 3 and 4, an anti-IDH1-R132H mAb HMab-1 recognized only IDH1-R132H peptide, indicating that HMab-1 is mono-specific. An anti-IDH1-R132S mAb SMab-1 reacted not only with IDH1-R132S but also with IDH1-R132V, IDH1-R132A, and IDH1-R132T, although IDH1-R132A and IDH1-R132T mutations have not been reported. RMab-3, an anti-IDH1 mAb, recognized not only all IDH1 mutations but also IDH2-R172C and IDH2-R172P, neither of which has been reported yet. In contrast, MsMab-1 reacted with almost all IDH1 mutations such as R132H and R132S except for R132W, R132V, R132P, and R132I. Furthermore, MsMab-1 reacted with 10 IDH2 mutations including R172S and R172G, both of which have been reported in gliomas or chondrosarcomas [3, 8]. Because mAbs against IDH2-R172S or IDH2-R172G have not been established, MsMab-1 is expected to be useful for the diagnosis of IDH2-R172S/R172G-bearing tumors.

**Table 2:** Synthetic IDH1 and IDH2 peptides for enzyme-linked immunosorbent assay

Gene	Mutation	Sequence
IDH1	WT	GGVKPIIIGRHAYGDQYRA
	R132H	GGVKPIIIGHHAYGDQYRA
	R132C	GGVKPIIIGCHAYGDQYRA
	R132S	GGVKPIIIGSHAYGDQYRA
	R132G	GGVKPIIIGGHAYGDQYRA
	R132L	GGVKPIIIGLHAYGDQYRA
	R132K	GGVKPIIIGKHAYGDQYRA
	R132M	GGVKPIIIGMHAYGDQYRA
	R132W	GGVKPIIIGWHAYGDQYRA
	R132V	GGVKPIIIGVHAYGDQYRA
	R132Q	GGVKPIIIGQHAYGDQYRA
	R132P	GGVKPIIIGPHAYGDQYRA
	R132I	GGVKPIIIGIHAYGDQYRA
	R132A	GGVKPIIIGAHAYGDQYRA
	R132Y	GGVKPIIIGYHAYGDQYRA
	R132D	GGVKPIIIGDHAYGDQYRA
	R132T	GGVKPIIIGTHAYGDQYRA
	R132E	GGVKPIIIGEHAYGDQYRA
	R132F	GGVKPIIIGFHAYGDQYRA
	R132N	GGVKPIIIGNHAYGDQYRA
IDH2	WT	GGTKPITIGRHAHGDQYKA
	R172H	GGTKPITIGHHAHGDQYKA
	R172C	GGTKPITIGCHAHGDQYKA
	R172S	GGTKPITIGSHAHGDQYKA
	R172G	GGTKPITIGGHAHGDQYKA
	R172L	GGTKPITIGLHAHGDQYKA
	R172K	GGTKPITIGKHAHGDQYKA
	R172M	GGTKPITIGMHAHGDQYKA
	R172W	GGTKPITIGWHAHGDQYKA
	R172V	GGTKPITIGVHAHGDQYKA
	R172Q	GGTKPITIGQHAHGDQYKA
	R172P	GGTKPITIGPHAHGDQYKA
	R172I	GGTKPITIGIHAHGDQYKA
	R172A	GGTKPITIGAHAHGDQYKA
	R172Y	GGTKPITIGYHAHGDQYKA
	R172D	GGTKPITIGDHAHGDQYKA
	R172T	GGTKPITIGTHAHGDQYKA
	R172E	GGTKPITIGEHAHGDQYKA
	R172F	GGTKPITIGFHAHGDQYKA
	R172N	GGTKPITIGNHAHGDQYKA

IDH, isocitrate dehydrogenase; WT, wild type.

**Table 3:** Reactivity of anti-IDH mAbs with mutated IDH1 peptides in ELISA

	WT	R132H^1^	R132C^1^	R132S^1^	R132G^1^	R132L^1^	R132K	R132M	R132W	R132V^1^	R132Q	R132P^1^	R132I	R132A	R132Y	R132D	R132T	R132E	R132F	R132N
MsMab-1	−	++	+	+++	+++	+	+	++	−	−	+++	−	−	+++	++	+++	+	+++	+	+++
HMab-1	−	+	−	−	−	−	−	−	−	−	−	−	−	−	−	−	−	−	−	−
SMab-1	−	−	−	+++	−	−	−	−	−	+	−	−	−	+++	−	−	+++	−	−	−
RMab-3	+++	+++	+++	+++	+++	++	+++	+++	++	+++	+++	+++	++	+++	+++	+++	+++	+++	++	+++

IDH, isocitrate dehydrogenase; WT, wild type; MsMab, multi-specific anti-mutated IDH1/2 mAb; ELISA, enzyme-linked immunosorbent assay; +++, OD655≥1.0; ++, 0.5≤OD655<1.0; +, OD655<0.5; −, negative.

1 These mutations have been reported.

**Table 4:** Reactivity of anti-IDH mAbs with mutated IDH2 peptides in ELISA

	WT	R172H	R172C	R172S^1^	R172G^1^	R172L	R172K^1^	R172M^1^	R172W^1^	R172V	R172Q	R172P	R172I	R172A	R172Y	R172D	R172T^1^	R172E	R172F	R172N
MsMab-1	−	−	++	+++	+++	++	−	++	−	−	+++	−	−	+++	++	+++	−	+++	+	−
HMab-1	−	−	−	−	−	−	−	−	−	−	−	−	−	−	−	−	−	−	−	−
SMab-1	−	−	−	−	−	−	−	−	−	−	−	−	−	−	−	−	−	−	−	−
RMab-3	−	−	++	−	−	−	−	−	−	−	−	++	−	−	−	−	−	−	−	−

IDH, isocitrate dehydrogenase; WT, wild type; MsMab, multi-specific anti-mutated IDH1/2 mAb; ELISA, enzyme-linked immunosorbent assay; +++, OD655≥1.0; ++, 0.5≤OD655<1.0; +, OD655<0.5; −, negative.

1 These mutations have been reported.

**Figure 2:**
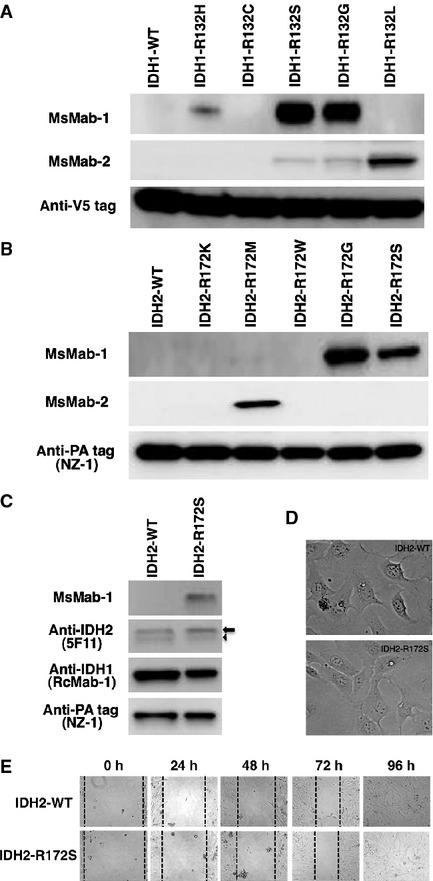
(A) Western blot analyses by MsMab-1 against mutated IDH1-expressing CHO cells. Total cell lysate from CHO cells expressing IDH1 wild type (WT, lane 1) and IDH1 mutants (lane 2, IDH1-R132H; lane 3, IDH1-R132C; lane 4, IDH1-R132S; lane 5, IDH1-R132G; lane 6, IDH1-R132L) were electrophoresed under reducing conditions using 5–20% gels, and were Western blotted with MsMab-1, MsMab-2, and anti-V5 tag. (B) Western blot analyses by MsMab-1 against mutated IDH2-expressing CHO cells. Total cell lysate from CHO cells expressing IDH2-WT (lane 1), IDH2 mutants (lane 2, IDH2-R172K; lane 3, IDH2-R172M; lane 4, IDH2-R172W; lane 5, IDH2-R172G; lane 6, IDH2-R172S) were electrophoresed under reducing conditions using 5–20% gels, and were Western blotted with MsMab-1, MsMab-2, and anti-PA tag (NZ-1). (C) Western blot analyses by MsMab-1 against mutated IDH2-expressing U-2 OS cells. Total cell lysate from U-2 OS cells expressing IDH2-WT (lane 1) and IDH2-R172S (lane 2) were electrophoresed under reducing conditions using 5–20% gels, and were Western blotted with MsMab-1, anti-IDH2 (5F11), anti-IDH1 (RcMab-1), and anti-PA tag (NZ-1). Arrow and arrowhead indicate transfected and endogenous IDH2, respectively. (D) The morphology of transfected U-2 OS cells (IDH2-WT and IDH2-R172S) was investigated 2 days after transfection. No morphological or growth difference was observed among those cells: 6.41 × 10^5^ cells and 6.56 × 10^5^ cells in 10-cm dish, respectively. (E) Measurement of cell migration by in vitro scratch assay. U-2 OS cells were transiently transfected with IDH2-WT or IDH2-R172S. The migration ability was assessed 24, 48, 72, and 96 h from the scratch. The figure shows the cells at 0, 24, 48, 72, and 96 h after the scratch. Magnification is 20×. IDH, isocitrate dehydrogenase; MsMab, multi-specific anti-mutated IDH1/2 mAb; CHO, Chinese hamster ovary; OS, osteosarcoma.

Western blot analyses were performed against mutated IDH1/2-expressing CHO cells. As presented in Figure 2A, MsMab-1 reacted strongly with V5 tag combined recombinant proteins of IDH1-R132S and IDH1-R132G expressed in CHO cells. It weakly recognized IDH1-R132H. It did not react with the other proteins (IDH1-WT [wild type], IDH1-R132C, IDH1-R132L), which indicates that MsMab-1 is useful for detecting IDH1-R132H/R132S/R132G expressed in mammalian cells. As presented in Figure 2B, MsMab-1 reacted strongly with PA tag combined recombinant proteins of IDH2-R172G and IDH2-R172S, reacted very weakly with IDH2-R172M expressed in CHO cells, but not with IDH2-WT or other IDH2 mutations. In contrast, another multi-specific anti-mutated IDH1/2 mAb, MsMab-2 did not react with IDH2-R172S. Furthermore, MsMab-1 detected IDH2-R172S expressed in U-2 OS human osteosarcoma cells (Fig. 2C), indicating that MsMab-1 is expected to be useful for immunohistochemical detection of IDH2-R172S osteosarcomas. Although IDH2-WT and IDH2-R172S were transfected into U-2 OS osteosarcoma cell line, no morphological or growth difference was observed among those cells 2 days after transfection (Fig. 2D). Furthermore, no cell migration difference was also detected between IDH2-WT and IDH2-R172S (Fig. 2E).

### Immunohistochemical analysis by MsMab-1 against IDH2-R172S-bearing osteosarcomas

**Figure 3:**
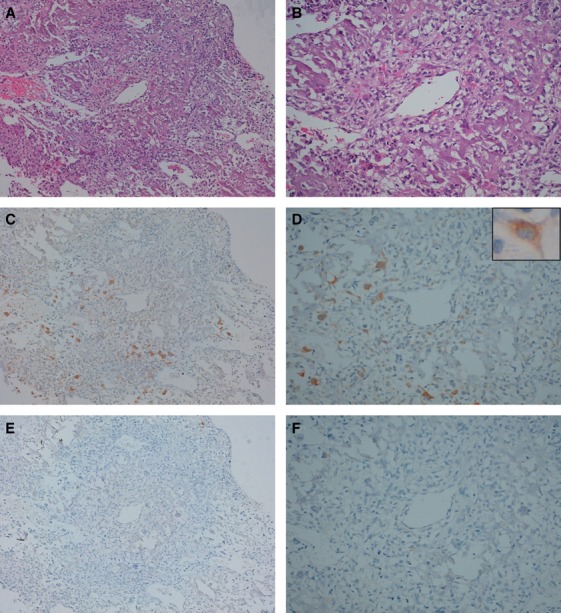
Immunohistochemical analyses by MsMab-1 against osteosarcoma tissues. (A and B) Hematoxylin-eosin staining against osteosarcoma tissues (OS10) possessing IDH2-R172S. (C and D) OS tissues were stained with MsMab-1. Insets show that MsMab-1 stained cytoplasm. (E and F) No staining was observed without a primary antibody. Magnification: (A, C, and E) 100×. (B, D, and F) 200×. MsMab, multi-specific anti-mutated IDH1/2 mAb; IDH, isocitrate dehydrogenase.

We next performed immunohistochemistry of MsMab-1 against IDH2-R172S-positive osteosarcomas (Fig. 3) because MsMab-1 strongly recognized IDH2-R172S proteins expressed in mammalian cells (Fig. 2B). MsMab-1 stained tumor cells of IDH2-R172S-positive osteosarcoma OS10 and OS11. However, it did not stain OS12 (Table 1; data not shown). Typical results are shown in Figure 3C and D. Some tumor cells were stained strongly by MsMab-1, although almost all tumor cells were stained moderately. MsMab-1 stained cytoplasm (inset of Fig. 3D). This cytoplasmic staining pattern of MsMab-1 is coincident with that described in previous reports [21, 22]. No staining was observed without primary mAb (Fig. 3E and F). MsMab-1 did not stain endothelial cells in osteosarcomas, indicating that MsMab-1 only stained mutation-bearing tumor cells (Fig. 3D). These results demonstrate that MsMab-1 is useful in immunohistochemical analyses for detection of IDH2-R172S mutations.

### Immunohistochemical analysis by MsMab-1 against tissue microarray of osteosarcomas

**Figure 4:**
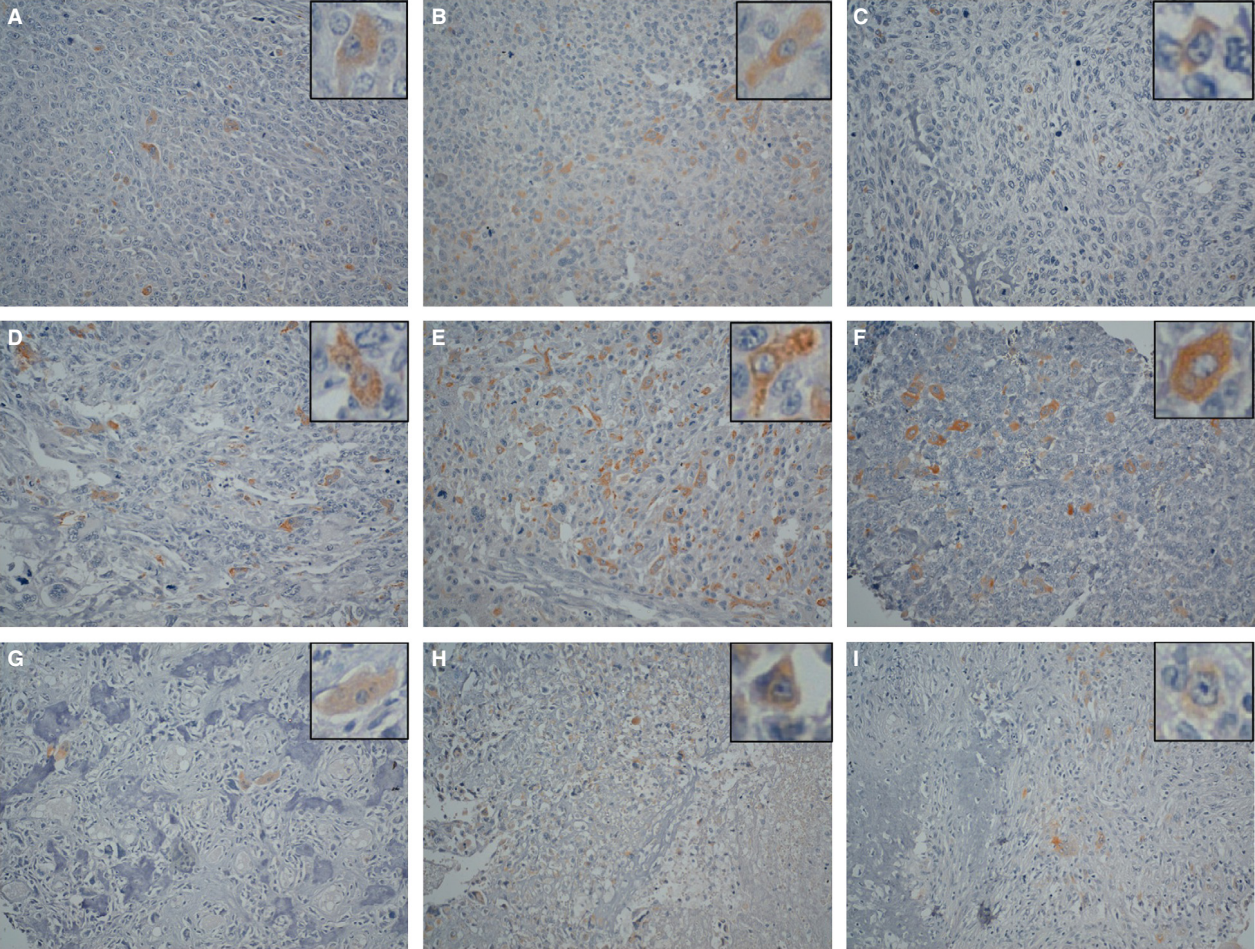
Immunohistochemical analysis by MsMab-1 against tissue microarray of osteosarcomas. Osteosarcoma tissue microarray was stained with MsMab-1 followed by LSAB kit. Color was developed using DAB, and was counterstained with hematoxylin. Typical results were shown: (A) No. 3, (B) No. 5, (C) No. 13, (D) No. 15, (E) No. 18, (F) No. 24, (G) No. 26, (H) No. 31, and (I) No. 32. Insets show that MsMab-1 stained cytoplasm (A–I). Magnification: 200×. MsMab, multi-specific anti-mutated IDH1/2 mAb; DAB, 3,3-diaminobenzidine tetrahydrochloride.

Finally, we performed immunohistochemistry against osteosarcoma tissue microarray using MsMab-1 mAb to investigate the expression of mutated IDH1/2. The characteristic of osteosarcoma patients is presented in Table 5. Because MsMab-1 reacted with several mutated IDH1/2 as confirmed by Western blot (Fig. 2), it is expected that MsMab-1 can show high sensitivity in immunohistochemistry. As shown in Table 5, MsMab-1 stained nine of 32 (28.1%) osteosarcomas. Typical staining patterns are portrayed in Figure 4. These results indicate that MsMab-1 is useful for immunohistochemical detection of mutated IDH1/2 in osteosarcomas.

**Table 5:** The characteristic of osteosarcoma patients (tissue microarray) used in immunohistochemical analysis by MsMab-1

					MsMab-1	
					
Patient no.	Age	Gender	Sample class	Diagnosis	Percentage^1^	Intensity^2^
1	69	M	Primary	OB	−	−
2	37	M	Primary	OB	−	−
3	62	F	Primary	OB	+	+++
4	29	M	Primary	OB	−	−
5	12	F	Primary	OB	++	+++
6	13	F	Primary	OB	−	−
7	17	M	Primary	CB	−	−
8	19	F	Primary	OB	−	−
9	15	F	Primary	OB	−	−
10	33	M	Primary	OB	−	−
11	12	M	Primary	OB	−	−
12	19	M	Primary	OB	−	−
13	16	M	Primary	OB	+	++
14	10	F	Primary	OB	−	−
15	14	F	Primary	OB	++	+++
16	31	F	Primary	OB	−	−
17	30	M	Primary	OB	−	−
18	18	M	Primary	OB	+++	+++
19	14	M	Primary	OB	−	−
20	43	M	Primary	OB	−	−
21	28	M	Primary	CB	−	−
22	10	M	Primary	OB	−	−
23	7	F	Primary	OB	−	−
24	37	F	Primary	OB	++	+++
25	34	M	Primary	OB	−	−
26	23	F	Primary	OB	+	++
27	14	F	Primary	OB	−	−
28	16	M	Primary	OB	−	−
29	60	M	Primary	OB	−	−
30	23	F	Primary	OB	−	−
31	16	M	Primary	OB	++	++
32	18	M	Primary	OB	+	++

MsMab, multi-specific anti-mutated IDH1/2 mAb; OB, osteoblastic osteosarcoma; CB, chondroblastic osteosarcoma.

1 −, no staining; +, <10%; ++, 10–50%; and +++, >50%.

2 −, no staining; +, weak; ++, medium; +++, strong.

## Discussion

This study demonstrated that osteosarcoma possesses IDH2 mutations, especially IDH2-R172S, which is also frequently observed with chondrosarcoma, which is another primary malignant bone tumor (Fig. 1). Amary et al. [8] reported that no IDH1/2 mutation was detected in non-cartilaginous neoplasms. In that report, osteosarcoma samples from 222 patients, 19 osteosarcoma cell lines, and one chordoma cell line were tested for IDH1 mutations using the high-throughput MassARRAY platform: 64.4% (143 samples) of these were also analyzed for IDH2 mutations, including all chondroblastic osteosarcomas. The discrepancy between our detection of IDH2 mutations in osteosarcomas and their analysis has not been resolved; it might be dependent on ethnic differences. The 12 patients for whom data are presented in Table 1 are all Japanese. The 32 patients in tissue microarray (Table 5) are all Chinese. In the near future, we expect to analyze IDH1/2 mutations using many more Japanese or Chinese osteosarcoma patients. Although we used subcloning method to confirm that OS12 possesses IDH2-R172S mutation (Fig. 1), MsMab-1 mAb did not stain OS12 in immunohistochemistry (Table 1), probably because (1) the sensitivity of MsMab-1 is not sufficiently high to detect all IDH2-R172S proteins in immunohistochemistry, (2) the expression of IDH2-R172S protein is heterogeneous in osteosarcoma tissues, as shown in Figures 3 and 4, or (3) IDH-R172S protein might be degraded in a paraffin section of OS12 because of fixation by formalin.

We previously established several anti-mutated IDH1/2 mAbs: HMab-1 against IDH1-R132H [14], SMab-1 against IDH1-R132S [16], GMab-r1 against IDH1-R132G [17], KMab-1 against IDH2-R172K [20], MMab-1 against IDH2-R172M [20], and WMab-1 against IDH2-R172W [21]. However, no mono-specific anti-IDH2-R172S mAb has been developed. Unexpectedly, we found that MsMab-1 detected IDH2-R172S, which was discovered in 25% of osteosarcoma patients in this study (Table 1). Moreover, MsMab-1 reacted with IDH2-R172G, which was reported in gliomas [3]. Because MsMab-1 did not react with IDH2-R172K or IDH2-R172W, and because it reacted weakly with IDH2-R172M, the combination of MsMab-1 with mono-specific mAbs such as KMab-1, MMab-1, and WMab-1 might still be necessary to detect all IDH1/2 mutations. For MsMab-1 reactivity, the discrepancy between ELISA (Tables 3 and 4) and Western blot analyses (Fig. 2) is expected to be resolved in the near future. MsMab-1 stained nine of 32 (28.1%) osteosarcomas (Fig. 4), indicating that MsMab-1 is extremely useful for the immunohistochemical detection of mutated IDH1/2 in osteosarcomas. The staining pattern of mutated IDH1/2 by MsMab-1 looks very heterogeneous (Fig. 4), which is different from that of glioma tissues [22]. Although we investigated 44 osteosarcoma patients in this study (Tables 1 and 5), we could not discuss the location of prepared tissues such as invasive or central portion, because we used biopsy samples from 12 patients (Table 1) and commercially available tissue microarray (Table 5). In a recent report, the staining pattern of IDH1-R132H by clone H09 mAb [19] in enchondroma of Ollier disease is heterogeneous, and the percentage of tumor cells positive for mutant IDH1 ranges from 50% to 90%, indicating intraneoplastic mosaicism [10]. Because intraneoplastic and somatic mosaicism has not been reported in osteosarcomas, the heterogeneity of MsMab-1 staining remains to be clarified.

Recently, two novel drugs were developed against mutated IDH1 and mutated IDH2. An inhibitor of mutant IDH1, AGI-5198, specifically suppressed the activity of mutant IDH1 and blocked the production of 2-HG [26]. Furthermore, AGI-5198 induced demethylation of histone H3K9me3 and expression of genes associated with gliogenic differentiation under conditions of 2-HG inhibition. Blockade of mutant IDH1 impaired the growth of glioma cells possessing mutant IDH1, but not that of IDH1 WT glioma cells without appreciable changes in genome-wide DNA methylation. Another inhibitor of mutant IDH2, AGI-6780, selectively inhibits the tumor-associated mutant IDH2-R140Q [27]. AGI-6780 induced differentiation of TF-1 erythroleukemia and primary human AML cells in vitro. Although we found IDH2-R172S of osteosarcomas in this study, no inhibitor against IDH2-R172S has been discovered. New agents must be developed to improve the survival of patients diagnosed with osteosarcoma [28], although several clinical trials have been conducted using various agents activated in osteosarcoma: imatinib, a tyrosine kinase inhibitor targeting platelet-derived growth factor (PDGF)-A against refractory or relapsed solid tumors [29]; sorafenib, a multikinase inhibitor targeting mitogen-activated protein kinase (MAPK), vascular endothelial growth factor receptors (VEGFRs), platelet-derived growth factor receptors (PDGFRs), and KIT against relapsed unresectable osteosarcomas [30]; trastuzumab, a mAb to HER2 against metastatic osteosarcoma patients [31]. Nevertheless, no dramatic effect has been observed. To improve the treatment of osteosarcoma further, drugs targeted against not only a molecular overexpression but also gene mutations such as mutated IDH1/2 must be developed.

Serum 2-HG is elevated in Ollier disease and Maffucci syndrome [9]. Dinardo et al. [32] recently reported that serum 2-HG levels predict IDH mutations and clinical outcome in AML. Their data confirm that serum measurement of an oncometabolite 2-HG provides useful diagnostic and prognostic information. The combination of immunohistochemistry using our developed anti-mutated IDH1/2 mAbs and serum measurement of an oncometabolite 2-HG is expected to improve osteosarcoma patient selection for IDH1/2-targeted therapy. Unfortunately, we could not check the 2-HG level before surgery, although we investigated 12 osteosarcoma patients. Serum measurement of 2-HG should be important in osteosarcomas; therefore, we will check the 2-HG before and after surgery of osteosarcoma patients in the next study.

It was reported that the potential for IDH mutations to produce 2-HG depends on allele specificity and subcellular compartmentalization [33]. The cellular 2-HG production from cytosolic IDH1 mutation is dependent on the activity of a retained WT IDH1 allele. The expression of mitochondrial IDH2 mutations led to robust 2-HG production in a manner that is independent of WT mitochondrial IDH function. Among the IDH2 mutations at R172 and R140, IDH2-R172 mutations consistently led to greater 2-HG accumulation than IDH2-R140 mutations did. From this perspective, it is important to elucidate the IDH mutations exactly in immunohistochemistry using mono-specific anti-mutated IDH1/2 mAbs such as HMab-1, SMab-1, GMab-r1, KMab-1, MMab-1, and WMab-1 as well as multi-specific mAbs MsMab-1 and MsMab-2. In the near future, IDH1/2 mutations will be acknowledged as useful diagnostic markers for osteosarcomas.
